# Supplementary material for: Uncovering specific mechanisms across cell types in dynamical models
Source: PLoS Comput Biol. 2023 Sep 13;19(9):e1010867. doi: 10.1371/journal.pcbi.1010867 (PMC10519600; doi:10.1371/journal.pcbi.1010867)
Supplement: S1 Text — (PDF) [file pcbi.1010867.s001.pdf]

**S1 Text: Comparison of common values for sensitivities**

The question of whether to use the mean or maximum of the corresponding sensitivities as a common value to ensure convergence of optimization was addressed in a simulation study with a workflow corresponding to the one depicted in Fig 3C. An ABC toy model is used

$$\dot{A} = -p_1 A$$
$$\dot{B} = p_1 A - p_2 B$$
$$\dot{C} = p_2 B$$

With the parameters $p_1, p_2$ and $init\_A$, representing the initial value for the state $A$, while the initial values of the other states are zero. Data was simulated for five cell-lines and regularization was performed with the standard LASSO and the symmetric penalization of fold-change differences (clustered LASSO) while using the maximum of sensitivities as a common value. The analysis was repeated with using the mean of sensitivities as a common value. The results indicate a better performance when using the mean (Supplemental Figure 3).
