# Supplementary material for: Uncovering specific mechanisms across cell types in dynamical models
Source: PLoS Comput Biol. 2023 Sep 13;19(9):e1010867. doi: 10.1371/journal.pcbi.1010867 (PMC10519600; doi:10.1371/journal.pcbi.1010867)
Supplement: S1 Fig — Objective function landscapes with the regularized best-fit parameter vector (red dot) for different regularization strengths λ for symmetric penalization of fold-change parameters with the L1 norm. The square bracket indicates a significant decrease in likelihood in terms of a likelihood ratio test. (PDF) [file pcbi.1010867.s002.pdf]

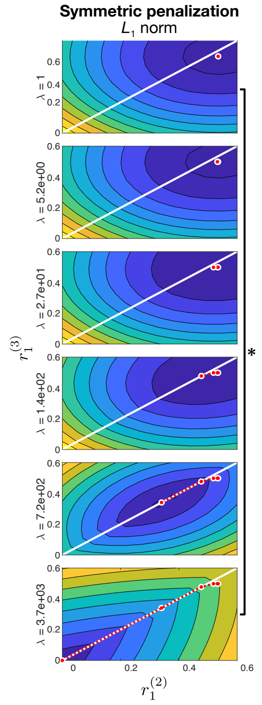

S1 Fig: Objective function landscapes with the regularized best-fit parameter vector (red dot) for different regularization strengths λ for symmetric penalization of fold-change parameters with the L_1 norm. The square bracket indicates a significant decrease in likelihood in terms of a likelihood ratio test.
