# Supplementary material for: Uncovering specific mechanisms across cell types in dynamical models
Source: PLoS Comput Biol. 2023 Sep 13;19(9):e1010867. doi: 10.1371/journal.pcbi.1010867 (PMC10519600; doi:10.1371/journal.pcbi.1010867)
Supplement: S2 Fig — (A) The number of cell type specific parameters dependent on the regularization strength in the model of [28] with symmetric penalization of fold-change differences. (B] The likelihood ratio (blue line) between the constrained and unconstrained model increases with the regularization strength. The largest value of λ for which the likelihood ratio is below the statistical threshold (red line) represents the parsimonious model (black line). The drop in the test statistic can be accounted to emergence of a new optimum. (C) Fold change parameters and differences of them with their values dependent on the regularization strength λ. Boxes denote the regions where a parameter is not constrained to zero, while the dashed line indicates the regularization strength corresponding to the parsimonious model. (PDF) [file pcbi.1010867.s003.pdf]

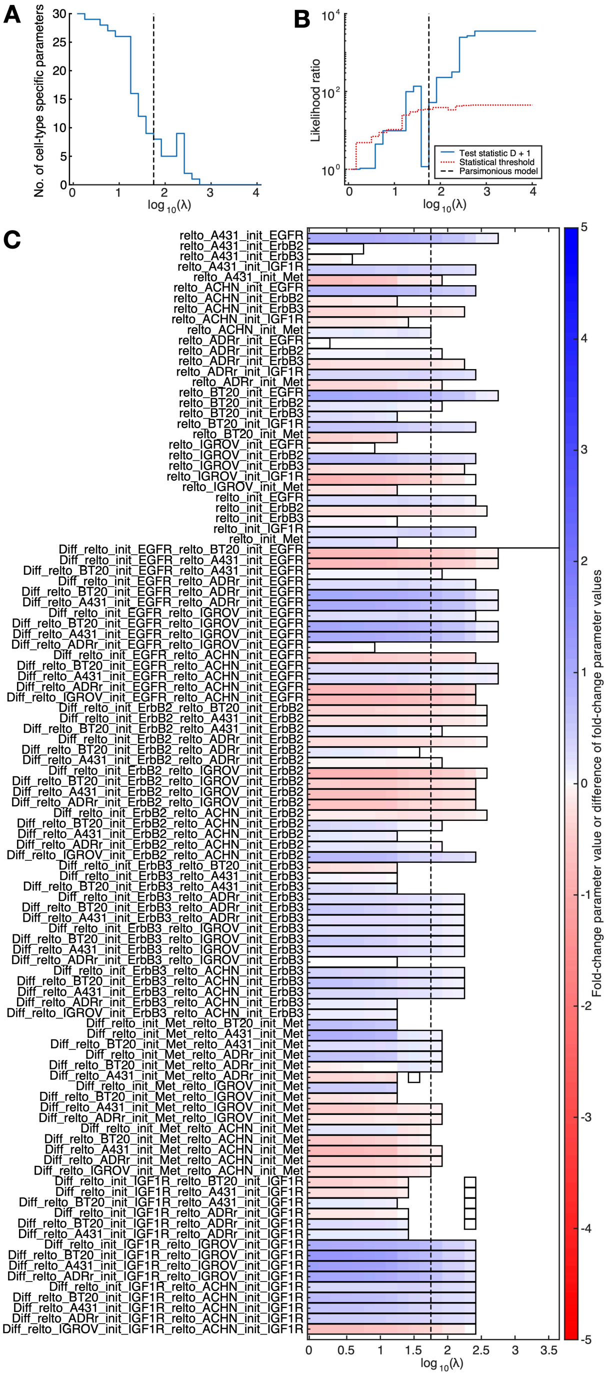

S2 Fig: (A) The number of cell type specific parameters dependent on the regularization strength in the model of [28] with symmetric penalization of fold-change differences. **(B)** The likelihood ratio (blue line) between the constrained and unconstrained model increases with the regularization strength. The largest value of λ for which the likelihood ratio is below the statistical threshold (red line) represents the parsimonious model (black line). The drop in the test statistic can be accounted to emergence of a new optimum. **(C)** Fold change parameters and differences of them with their values dependent on the regularization strength λ. Boxes denote the regions where a parameter is not constrained to zero, while the dashed line indicates the regularization strength corresponding to the parsimonious model.
