# Supplementary material for: Uncovering specific mechanisms across cell types in dynamical models
Source: PLoS Comput Biol. 2023 Sep 13;19(9):e1010867. doi: 10.1371/journal.pcbi.1010867 (PMC10519600; doi:10.1371/journal.pcbi.1010867)
Supplement: S3 Fig — (PDF) [file pcbi.1010867.s004.pdf]

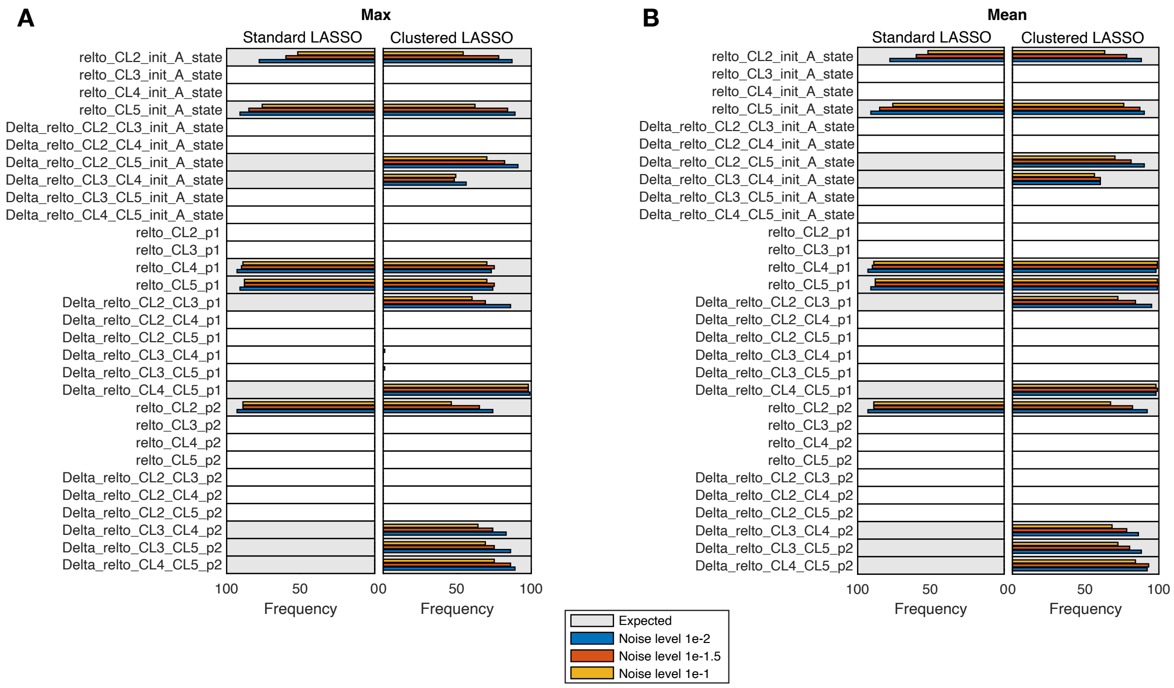

S3 Fig: Simulation study (Fig 3C) with an ABC model to assess the performance of regularization with symmetric penalization of fold-change differences when using **(A)** the maximum of sensitivities corresponding to the same residual as a common value, and **(B)**, the mean of sensitivities as a common value.
