# Supplementary material for: Uncovering specific mechanisms across cell types in dynamical models
Source: PLoS Comput Biol. 2023 Sep 13;19(9):e1010867. doi: 10.1371/journal.pcbi.1010867 (PMC10519600; doi:10.1371/journal.pcbi.1010867)
Supplement: Attachment — Submitted filename: answer.pdf [file pcbi.1010867.s007.pdf]

Dear Prof. Dr. Gabhann, dear Prof. Dr. Papin,

Thank you for considering our manuscript „Uncovering specific mechanisms across cell types in dynamical models", as well as collecting and reviewing the reviewer's comments. We also appreciate the extension of the resubmission deadline you granted us with.

Please find enclosed our point-by-point reply to the comments made by the reviewers. We hope the reviewers find our response to their questions satisfactory. We also attached a revised version of our manuscript with the changes highlighted.

We hope that it is now possible for you to consider the publication of our work to the broad readership of PLOS Computational Biology.

Yours sincerely,

Dr. Adrian L. Hauber
Institute of Physics, Univ. of Freiburg
Hermann-Herder-Str. 3a
79104 Freiburg im Breisgau
Germany

E-Mail: adrian.hauber@fdm.uni-freiburg.de
Phone: +49 761 2037639

Reviewer 1

This is indeed a pending issue in the field, and an original contribution. The paper then applies the new method to the determination of cell-specific parameters in a toy model and in simulated data.

> We would like to mention that the proposed method is also applied to experimental data from Hass et al. 2019.

The authors state in several places, notably the abstract, that the current methods are not consistent biologically for n>2. This is not substantiated. While mutations and CNVs are discrete by nature, the assumption that the distribution of true parameter values in a population

> We thank the reviewer for their remark concerning the consistency of existing methods for n > 2 cell types and would like to refer to the section „The challenge of n>2 cell types" for a discussion on the problems that potentially arise when applying existing methods to more than two cell types.
>
> In many applications we observed continuous differences, e.g. in protein abundance https://www.mdpi.com/2072-6694/14/10/2379/htm
>
> For further clarification on the applicability of different regularization methods, we added Table 1 to our revised manuscript.

[line 139] This is misleading… ref 20 (De Landtsheer et al.) uses a regularization term that also does not depend on the choice of reference cell line and also performs supervised clustering, not the L1 norm. In addition, that paper does not use ODEs. Also, ref 24 (Raimundez et al.) explicitly states they used an iterative method instead of the L1 norm of ref 12 (Steiert et al.) which they tried and concluded it did not work. These two papers should be discussed further.

> We thank for this comment and agree with the reviewer that the references Raimundez et al. and De Landtsheer et al. do not fit perfectly in this context. Since Raimundez et al. state that they tried the method and it gave no meaningful results in their case which can have many

particular reasons, we decided to remove this reference. As for the Landtsheer et al. reference we added the information that the penalty is here used in the context of logical modeling. We as well added another new reference (Lao-Martil et al.) that uses the L_1 penalty and fits better.

[line 142] The value "0.8" is from the paper by Dolejsch et al., and is inferred from a small dataset. Authors should specify why they think this specific value is appropriate in their case. Ref 25 (Turia et al.), while mentioning non-convex optimization, is an unrelated paper about image processing, using the GIST algorithm.

We agree that it is of potential interest to analyze which norm is the best with respect to different classes of ODE models, but this was not in the scope of this paper. While the value of $q = 0.8$ does only pose a heuristic, it results in general in a better regularization performance compared to the standard LASSO with $q = 1$, as discussed in Dolejsch et al. 2019. We therefore applied this value also for the clustered LASSO which also resulted in a faster identification of the sparsity structure (c.f. Figures 2D and S1). We removed reference 25 as suggested by the reviewer.

[lines 150-155] The obvious solution to the stated problem would be to average over all possible choices of reference cell type. The authors should specify if their method is a formulation of this obvious solution or in which aspect it differs.

We thank the reviewer for their remark concerning an alternative approach for handling the case of $n>2$ cell types. We want to emphasize that the result of the regularization is a yes-no-decision for the parameter values being the same or not and summing up does not make sense in this regard. However, in fact, the symmetric penalty corresponds to averaging over the penalties of all potential reference cell types and dividing by two. Since we agree that this is an important insight, we amended section III accordingly.

[lines 222-225]: For ri(j) – ri(k) the gradient of equation 5 is zero, the divergence is an artifact of the sign function. The 10E-10 threshold seems to be instead the standard way to avoid numerical errors in computations. Please clarify this point.

We thank the reviewer their remark and would like to emphasize that the divergence is due to the expression $|x|^q$ with $x = 0$ and $q < 1$. Choosing a threshold of 10E-10 is in accordance with Dolejsch et al. 2019. We reformulated the corresponding sentence in the revised manuscript to make this clearer.

[lines 258-259]: parameters have been defined as strictly positive on line 107, so there is no sign change. The authors might have omitted the word 'differences' before the comma.

In ll. 258-259, sign changes of the difference of fold-change parameters $|r_i^j - r_i^k|$ are discussed. We would like to point out that while $\tilde{r}_i^j$ is indeed strictly positive, r_i^j is not since $r_i^j = \log \tilde{r}_i^j$ (Equation 2).

[line 283 and others]: The authors make use of a likelihood ratio test with alpha = 0.05. It would be much better to provide the p-values. The authors should also state why they use such test instead of the more commonly used information criteria (AIC, BIC).

We thank the reviewer for their point concerning the model discrimination technique employed for identifying the parsimonious model. We employed a likelihood ratio test since it poses a solid statistical method and was already used in the work of Steiert et al. 2016, where L1 regularization was established as a method within dynamical modeling. We added an explanation on the choice of the statistical method in a revised manuscript. We did not provide p-values since it is a yes/no decision.

[figure 2B]. Either "p1" should be "log(p1)", or the sign should be changed in the table. The double negative sign makes the exponential increase instead of decrease.

We thank the reviewer for pointing out this mistake and changed p1 to log(p1) in the revised manuscript.

[Figure 2D]. The text on line 338 indicates 6 values of lambda, but the figure only shows 5. The y axis should be indicated on the plots on the right side. I think it is possible to merge the figures from the supplementary data in here (L1 landscape and plots of the objective function and number of parameters versus lambda), as it would help comparison. Refering to line 350, indicating the true value of the parameters on the plots might help the reader to evaluate the bias induced by the regularization. It seems that the third plot on the right is the most optimal one, as the values for r2 and r3 are equal and the bias is minimal, however it is not clear what is the result of the statistical procedure. Here also, indicating p-values might be more appropriate than a "".*

We thank the reviewer for their feedback concerning Figure 2D. We removed inconsistencies in the text related to the number of values for λ, and added a y-axis to the right column of plots, as suggested by the reviewer. We did however not merge Figure 2D with the corresponding Figure in the Supplementary Information due to space limitations and to facilitate readability.

Concerning the suggestion of showing the true values of the parameters $r^{(2)}$ and $r^{(3)}$, we would like to point out that Figure 2D portrays the optimization landscape in the regularized setting, where optimization endpoints will in general not converge to the true values of parameters, but include some degree of bias. As described in l. 273 ff., we conduct a unregularized, constrained optimization afterwards which includes no bias. Therefore, we consider inspection of the bias on parameter estimates not to be relevant at this point.

We therefore decided not to include the true values in this plot, as we think it would be more confusing than helping with understanding the figure.

We decided against using p-values as it would introduce additional complexity to the already information-dense plot, which seems unnecessary to us, as it portrays simulated data only, for which p-values are not interesting. To make the figure more understandable, we included shaded areas that denote the corresponding model structures, which are tested against each other.

[line 363] I don't see where the value lambda=27 comes from

We thank the reviewer for pointing out this mistake and changed the value of λ to the correct value in the revised manuscript.

[Figure 3] Please add a schematic representation of the ODE system to help readers understand which parameters are expected to be cell-line specific. There is a typo ("penlization") and it's not clear why values for k_on have more decimals than the others.

We thank the reviewer for their remark. We added a schematic representation of the model to Fig. 3, corrected the typo, and made the decimals of "k_on" consistent.

[line 432]: this statement needs a reference.

We thank the reviewer for their remark and added a reference.

[line 438]: the authors should clarify why they mention which cell line is the "technical" reference while stating earlier that is has no importance, as it might confuse readers.

We thank the reviewer for their remark and removed the mention of H322M as the technical cell line as it is a mere technical detail that might confuse readers.

[figure 4]: this figure is hard to interpret. I suggest using simple scatterplots (model estimate versus receptor abundance, two series) and reporting how both models correlate with the CCLE-inferred values.

We thank the reviewer for their feedback concerning figure 4. However, we do not think that using scatter plots would increase interpretability of this figure. We specifically chose this layout to emphasize the convergence of receptor abundances under different regularization techniques, and not their numerical values, which the plot in its current form nicely portrays.

Ref 16 by Yiyuan She is the well-known paper formalizing the clustered lasso. The proposed method uses both grouped and clustered regularization. As per the cited paper: "Grouped lasso assumes the grouping of features (predictors) is known, arising naturally from the underlying background, such as the dummy variables introduced for a multi-level factor. The coefficients within the same predictor group are not necessarily equal. The clustered lasso performs supervised clustering and groups the predictors taking into account both X and y". That is what "She" said. The authors should clarify these two notions, as the application by She concerns groups of genes without a priori clustering. Also, that paper establishes that the naïve clustered LASSO is not optimal and proposes a series of improvements, this could be discussed or at least in the paper.

We thank the reviewer for their remark. Concerning the different regularization approaches "grouped" and "clustered", we added Table 1 to the manuscript to better show applicability of these in different contexts of ODE modeling. Furthermore, we added a discussion of the limitations of the clustered LASSO to the revised manuscript. We are aware of the several improvements on L1 regularization suggested by She et al., however, to test all of these in the context of ODE modeling was beyond the scope of our work. Therefor, by using the $L_{0.8}$ penalty we followed Dolejsch et al. which is to the best of our knowledge the best benchmark study in the context of ODE modeling.

The authors should specify, either in the paper or on the repository, which version of Matlab is required, as well as the necessary Add-ons.

We thank the reviewer for their remark and added a more specific description of the Matlab version that was used in the revised manuscript.

Reviewer 2

From Figure 1b, it is clear that when Lambda is sufficiently large, the parameter estimation process will strongly favor maximizing the number of parameters with identical values due to symmetric penalization. Although this is the main reason for implementing this form of regularization, it can lead to the identification of multiple false positives, such as several mutations that are identified to be common but are not. This is especially probable in underdetermined problems, where parameters can vary widely due to the scarcity of observations, as noted by Gutenkunst et al (PloS Comp Bio, 2007). Since the authors focus on biological systems as examples, which generally lack sufficient observations compared to the number of parameters that need to be identified, this issue should be explicitly mentioned and discussed. Can we avoid this problem at all?

We agree with the reviewer that within all modeling approaches, especially in non-linear ODE modeling with sparse data, drawing wrong conclusions is a problem. Mathematical models of

biological systems are on the other side very powerful to inform where and what to measure in order to answer biological questions. Additionally, a powerful approach is to examine the profile likelihood of the fold-change parameters after regularization to avoid false positives, as discussed by Steiert et al. 2016. In general, it is never easy to draw conclusions from a model without biological validation. We added a new paragraph to the conclusions section.

Related to my previous remark, in the presented toy example, it is possible for cell types 2 and 3 to have distinct mutations, yet the proposed algorithm might identify them as common. Moreover, since clustered LASSO does not yield the actual parameter values (instead, it tends to shrink them toward zero, like standard LASSO), it is unclear why one should apply this regularization in such a scenario. In general, the authors should provide clear guidance to readers on when it is appropriate and when it is not advisable to use their regularization method.

We thank the reviewer for their remark and would like to point out that we propose to apply a two-step procedure that includes the application of the clustered LASSO regularization to identify candidate model structures that exhibit common mutations across cell lines, as well as a subsequent unregularized but constrained parameter estimation to circumvent the unwanted effect of shrinkage. For details, see l. 273ff of the original manuscript. We would like to point out that the identification of identical mutations in cell type 2 and 3 is due to the small amount of data compared to the difference in the values of p. We chose this setting to make the example more illustrative. We agree with the reviewer in that it is hard for the reader to grasp the applicability of the different regularization methods, which is why we added Table 1 to the revised manuscript.

Given that linear correlations between parameters are typical in various systems, I would like to know if this regularization method can be extended to address such cases. I assume that the correlation coefficients would probably appear as the optimization variables in the penalty term. Can you comment on this?

We thank the reviewer for raising this point. Linear correlations between parameters are indeed problematic for our method, as it is for the simpler case of N=2. To address this point, we have been testing different approaches within our group. One (conceptually known) idea is to add an additional L_2 prior to the parameters, or to use an $L_{0.8}$ prior instead of L_1, as was discussed in Dolejsch et al. and as we therefor also do here. Most of the problems coming from linear correlation should be solved by this. The reviewer's idea of adding the correlation coefficient to the objective function is interesting, however, within a single computation of the objective function, it is unclear how to properly define correlations between parameters, since they are only apparent between parameter sets of different evaluations of the objective function.

The explanation in lines 310-320 and Figure 2 could be clearer regarding the toy model. It is hard to distinguish what the authors consider as reality and what is meant to provide "realistic" data for the example. Moreover, in the text, the species is denoted with x, in Figure as A. The value for parameter p in the text is logp=-1.5 whereas in Figure 2b we find -2 (I was trying to deduce based on the graph in Figure 2c what was used). Next, the values in Figure 2b are not p (as written at the top of the table), these should be log p. Similarly in Figure 2A, you have a mix of notations. With the notation presented earlier in the manuscript, I would not say that you can write p1r1^(2) and p1r1(3).

We thank the reviewer for making us aware of the inconsistencies concerning Figure 2, which we accounted for by updating the figure and the corresponding section in the manuscript.

Regarding the size of systems this kind of regularization can be used on, how does this method scale computationally when the number of cell types increases and the number of parameters? Please provide the computational time estimates.

Thanks for this question. Comparing computation times of parameter estimation is always complicated without proper benchmarking. Within maximum-likelihood estimation in ODE models, the bottleneck in computation time lies in the integration step that has to be repeated after each optimization step. Concerning multiple cell types, the integration time that is necessary is multiplied by the number of cell types (similar as if multiple experimental conditions need to be integrated), independent from any further penalty term on the objective function. In other words, the evaluation time of the objective function is not affected by the additional regularization terms. It might of cause happen that the number of optimization steps increases which also affects the overall fitting time, but this is hard to compare, since it depends on a lot of other factors such as starting position in parameter space, stiffness of the ODE, and settings of the integrator and optimizer. This is the reason why we decided not to report computation time estimates. To make this point clearer in the manuscript, we amended the conclusions section accordingly.

Since this regularization is proposed in the context of nonlinear optimization, how can you guarantee you found a satisfying result? Furthermore, can you enumerate all alternative solutions of clustered parameters? For example, in the study depicted in Figure 4, there are cell line groups that have the same parameter values. Will these groups change if you converge on a different solution?

We agree with the reviewer that, generally, when applying LASSO based methods in nonlinear ODE modeling, interpretation of results is challenging. It is generally not possible to enumerate all solutions and parameter groups might change depending on the starting point of the optimization. One way to circumvent this, is to apply a multi-start optimization for all regularization strengths. First of all this is computationally very expensive, and also with this approach, one cannot be sure to have „the correct“ solution. Results are in a sense always just a hint what to validate biologically. The scope of our work was to extend the existing methodology from the case N=2 to the cases N>2. Therefore, we regard the question of how to find the global optimum in parameter space in the setting of dynamical modeling as beyond the scope of this paper. To address this point and the reviewer’s first point we added a general paragraph on limitations of the method to the Conclusions section.

Lines 240-250, to avoid that r_i^(j)-r_i^(k)=0, r_i^(j) <>0 occurs, would it work using a kind of forgetting factor?

Unfortunately, we are not able to completely understand what the reviewer means by a forgetting factor in this case. If we are not mistaken, forgetting factors would imply that the optimization routine is no longer deterministic, but this would mean additional problems for the interpretation of the optimization.

The notation throughout the paper is confusing. For example, lines 120-126, one understands that in r_i^(j) the subscript i denotes the i-th parameters, whereas the superscript j denotes the cell type. But then, lines 199-200, we see the same subscript i denoting “the l-th and the o-th parameter”. Furthermore, lines 210-212, what the subscript l-n denotes. In general, for most superscripts and subscripts, their range is missing.

We thank the reviewer for their remark concerning the notation of fold-change parameters. We would like to point out the switch in notation described in l. 199 of the original manuscript that is necessary because the technical aspects are much easier explained by using a numbering scheme for parameters that just counts them with only one index, while neglecting their mechanistic meaning. However, for introducing the fold-change parameters, we chose a two-index notation $r_i^{(k)}$, which relates the fold-change parameter to the corresponding parameter p_i, as well as the cell type k.

We thank the reviewer for pointing out the missing explanation concerning the subscript "l-n" in l. 212, which we added in the revised manuscript.

Line 347, there is a floating sentence fragment.

We thank the reviewer for their remark and incorporated a fix for the typo in our revised manuscript.

Reviewer 3

The authors provide three examples, illustrating the application of their proposed regularization approach, ranging from fully simulated to completely experimental, where an experiment by Hass et al. (2017) is repeated, incorporating the proposed grouped LASSO approach. A major concern, however, relates to the method of hyperparameter tuning. In the proposed approach, selection of the hyperparameter occurs by performing a likelihood ratio test on the fitted likelihood values. While the approach taken seems justified in general, the likelihood ratio test should instead be performed on likelihood values calculated on samples unseen during the parameter estimation procedure. This prevents biasing the results towards the specific samples included in the data. This reviewer recommends including a cross-validation approach to strengthen the hyperparameter selection.

We thank the reviewer for the suggestion of examining different methodologies of finding the correct hyperparameter λ. Determining hyperparameters by unseen data is typically not possible in the field of our applications, since data are rather limited. Moreover the likelihood ratio test is by construction (asymptotically) valid for in sample data.

Instead, we interpret the hyperparameter λ as an auxiliary variable that by variation suggests different meaningful model structures (including different sparsity) that have to be tested against each other. In that sense, a simple model selection criterion is necessary which is given for example by the likelihood ratio test.

In the explanation on parameter estimation in dynamical systems, the authors mention that parameters are inferred from normally distributed data. It seems that the authors may have meant normally distributed error in the data, as the measurements that are used to fit the dynamic model are not at all normally distributed.

We thank the reviewer for pointing out this mistake and corrected it in our revised manuscript.

In the section Adaptations to the optimization algorithm, two parameters are introduced with subscripts l and o in the first subsection, while in the subsequent subsections, these parameters seem to be referred to using subscripts l and n. Please improve the consistency in the notation.

We thank the reviewer for their remark. We incorporated the suggested changes into our revised manuscript.

In the formulae for the residual and the sensitivity of the residual in the same section as in the previous comments, the norm is divided by 1/lambda, which could be made clearer by multiplying the norm with lambda instead.

We thank the reviewer for their suggestions and included them into our revised manuscript.
