# Supplementary material for: Uncovering specific mechanisms across cell types in dynamical models
Source: PLoS Comput Biol. 2023 Sep 13;19(9):e1010867. doi: 10.1371/journal.pcbi.1010867 (PMC10519600; doi:10.1371/journal.pcbi.1010867)
Supplement: Attachment — Submitted filename: ReviewPlosCompBio202303.docx [file pcbi.1010867.s006.docx]

Uncovering specific mechanisms across cell types in dynamical models

Hauber et al.

The paper describes a new methodology to smooth out the overfitting of ODE systems, when using data taken from more than two biological contexts. The authors adapted the clustered LASSO to the optimization formalism most used when training ODE systems, in the form of a regularization term in the objective function. This is indeed a pending issue in the field, and an original contribution. The paper then applies the new method to the determination of cell-specific parameters in a toy model and in simulated data. An implementation is provided in the D2D Matlab toolbox, as well as a demo script.

Language and grammar are appropriate, and the organization of the paper makes sense. The code runs as described. However, there is a set of important issues that need to be revised:

1) The authors state in several places, notably the abstract, that the current methods are not consistent biologically for n>2. This is not substantiated. While mutations and CNVs are discrete by nature, the assumption that the distribution of true parameter values in a population is never continuous should be explicitly discussed in the introduction.
2) [line 139] This is misleading… ref 20 (De Landtsheer et al.) uses a regularization term that also does not depend on the choice of reference cell line and also performs supervised clustering, not the L1 norm. In addition, that paper does not use ODEs. Also, ref 24 (Raimundez et al.) explicitly states they used an iterative method instead of the L1 norm of ref 12 (Steiert et al.) which they tried and concluded it did not work. These two papers should be discussed further.
3) [line 142] The value “0.8” is from the paper by Dolejsch et al., and is inferred from a small dataset. Authors should specify why they think this specific value is appropriate in their case. Ref 25 (Turia et al.), while mentioning non-convex optimization, is an unrelated paper about image processing, using the GIST algorithm.
4) [lines 150-155] The obvious solution to the stated problem would be to average over all possible choices of reference cell type. The authors should specify if their method is a formulation of this obvious solution or in which aspect it differs.
5) [lines 222-225]: For ri(j) – ri(k) the gradient of equation 5 is zero, the divergence is an artifact of the sign function. The 10E-10 threshold seems to be instead the standard way to avoid numerical errors in computations. Please clarify this point.
6) [lines 258-259]: parameters have been defined as strictly positive on line 107, so there is no sign change. The authors might have omitted the word ‘differences’ before the comma.
7) [line 283 and others]: The authors make use of a likelihood ratio test with alpha = 0.05. It would be much better to provide the p-values. The authors should also state why they use such test instead of the more commonly used information criteria (AIC, BIC).
8) [figure 2B]. Either “p1” should be “log(p1)”, or the sign should be changed in the table. The double negative sign makes the exponential increase instead of decrease.
9) [Figure 2D]. The text on line 338 indicates 6 values of lambda, but the figure only shows 5. The y axis should be indicated on the plots on the right side. I think it is possible to merge the figures from the supplementary data in here (L1 landscape and plots of the objective function and number of parameters versus lambda), as it would help comparison. Refering to line 350, indicating the true value of the parameters on the plots might help the reader to evaluate the bias induced by the regularization. It seems that the third plot on the right is the most optimal one, as the values for r2 and r3 are equal and the bias is minimal, however it is not clear what is the result of the statistical procedure. Here also, indicating p-values might be more appropriate than a “*”.
10) [line 363] I don’t see where the value lambda=27 comes from.
11) [Figure 3] Please add a schematic representation of the ODE system to help readers understand which parameters are expected to be cell-line specific. There is a typo (“penlization”) and it’s not clear why values for k_on have more decimals than the others.
12) [line 432]: this statement needs a reference.
13) [line 438]: the authors should clarify why they mention which cell line is the “technical” reference while stating earlier that is has no importance, as it might confuse readers.
14) [figure 4]: this figure is hard to interpret. I suggest using simple scatterplots (model estimate versus receptor abundance, two series) and reporting how both models correlate with the CCLE-inferred values.
15) Ref 16 by Yiyuan She is the well-known paper formalizing the clustered lasso. The proposed method uses both grouped and clustered regularization. As per the cited paper: “Grouped lasso assumes the grouping of features (predictors) is known, arising naturally from the underlying background, such as the dummy variables introduced for a multi-level factor. The coefficients within the same predictor group are not necessarily equal. The clustered lasso performs supervised clustering and groups the predictors taking into account both X and y”. That is what “She” said. The authors should clarify these two notions, as the application by She concerns groups of genes without a priori clustering. Also, that paper establishes that the naïve clustered LASSO is not optimal and proposes a series of improvements, this could be discussed or at least mentioned in the paper.
16) The authors should specify, either in the paper or on the repository, which version of Matlab is required, as well as the necessary Add-ons.
